# ERgene: Python library for screening endogenous reference genes

**DOI:** 10.1038/s41598-020-75586-5

**Published:** 2020-10-29

**Authors:** Zehua Zeng, Yuzhe Xiong, Wenhuan Guo, Hongwu Du

**Affiliations:** 112 Lab, School of Chemistry and Biological Engineering, University of Science and Technology Beijing, Beijing, 100083 China

**Keywords:** Bioinformatics, Genomic analysis

## Abstract

In gene expression analysis, sample differences and experimental operation differences are common, but sometimes, these differences will cause serious errors to the results or even make the results meaningless. Finding suitable internal reference genes efficiently to eliminate errors is a challenge. Aside from the need for high efficiency, there is no package for screening endogenous genes available in Python. Here, we introduce ERgene, a Python library for screening endogenous reference genes. It has extremely high computational efficiency and simple operation steps. The principle is based on the inverse process of the internal reference method, and the robust matrix block operation makes the selection of internal reference genes faster than any other method.

## Introduction

Gene expression analysis has become increasingly important in many areas of biological research. The commonly used measurement methods include microarray^1^, RT-PCR^2^ and massively parallel sequencing^3^. However, these measurements also require normalization to reduce the differences between samples. The existing normalization methods include geNorm^4^, Normfinder^5^ and BestKeeper^6^. All three methods start with a limited set of candidate reference genes. Further, geNorm and BestKeeper also calculate a normalization factor. On the one hand, the calculation efficiency of the above methods is not high enough, and the screening process is sometimes complicated. On the other hand, there are currently no available package for screening endogenous reference genes in Python. In order to solve these problems, a new approach is proposed by analyzing the principle of the internal reference method. Using the computational power of the Pandas library in Python, we build a Python library to meet the requirements of normalization and internal reference gene screening.

## Results

### Screening effect of laboratory gene expression data

**Figure 1:**
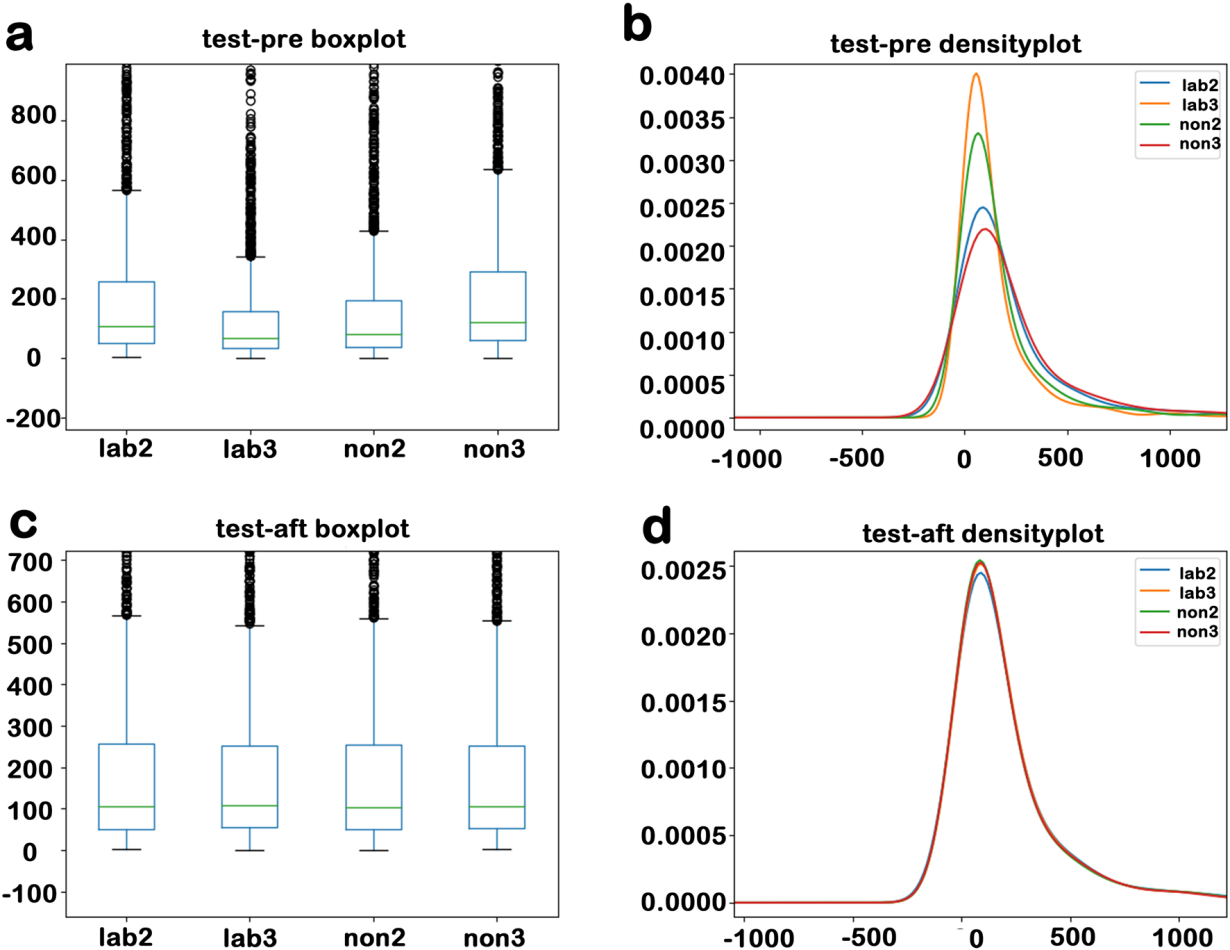
(**a**) The boxplot of test data before processing. (**b**) The density plot of the test data before processing. (**c**) The boxplot of test data after processing. (**d**) The density plot of the test data after processing. The boxplot’s abscissa is the sample, the ordinate is the gene expression, the green line is the median, the blue box line is the quartile, and the black point is the outlier. The density plot’s abscissa is the length of the data, the ordinate is the data density, and the lines of different colors represent different samples.

We took some tissues from the same location in the brains of two aging mice injected with SHED (Stem cells from human exfoliated deciduous teeth) and two aging mice injected with salt. And gene expression analysis was performed on these tissues to obtain test data. The test data can be found on Github (https://github.com/Starlitnightly/ERgene/tree/master/example). First, we analyzed the difference of the sample spectral density in the test data and made a box and a density diagram (Fig. 1a,b). The difference of the sample spectral density generally refers to differences in the spectral density of all gene expressions between each sample, such as the differences between individual mice, or the differences between experiments. If the difference of the sample spectral density is too large, the subsequent analysis will be meaningless. To avoid this, researchers typically use internal reference genes to normalize the data. Therefore, it is very important to look for stable internal reference genes. In the test data, we used the ERgene.FindERG method. Then the candidate internal reference gene Atp1a3 was found. Using the gene Atp1a3, we normalized the test data and made the boxplot in the same way (Fig. 1c,d). By comparing Figs. 1a,c and Fig. 1b,d, we can see that the difference of the sample spectral density has been significantly reduced. Therefore, ERgene has a significant effect on reduce the difference of the sample spectral density when it comes to processing raw lab data.

### Screening effect of public datasets

**Figure 2:**
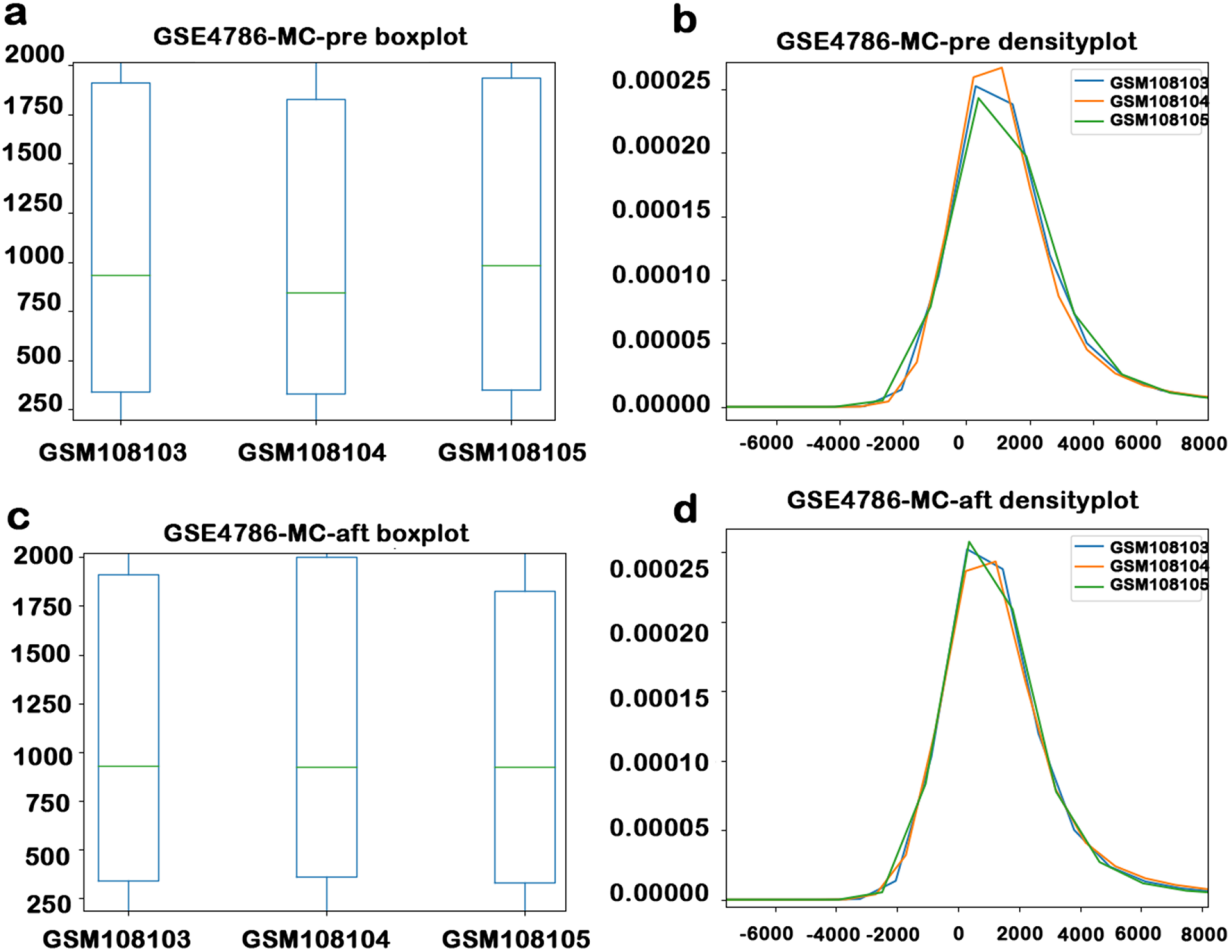
(**a**) The boxplot of GSE4786-MC before processing. (**b**) The density plot of the GSE4786-MC before processing. (**c**) The boxplot of GSE4786-MC after processing. (**d**) The density plot of the GSE4786-MC after processing. The boxplot’s abscissa is the sample, the ordinate is the gene expression, the green line is the median, the blue box line is the quartile, and the black point is the outlier. The density plot’s abscissa is the length of the data, the ordinate is the data density, and the lines of different colors represent different samples.

After obtaining good results from the raw lab test data, we selected a dataset in the GEO database that had not been well normalized for verification. The dataset selected was the mouse dataset GSE4786 of Someya^7^. We analyzed the difference of the sample spectral in the experimental data and made a box diagram and a density diagram (Fig. 2a,b). Then, we used the ERgene.FindERG method and found the internal reference gene 1439423_x_at. Using the gene 1439423_x_at, we normalized the data and plotted the boxplot in the same way (Fig. 2c,d). By comparing Figs. 2a,c and Fig. 2b,d, we can see that the difference of the sample spectral has been narrowed. The reason for choosing this dataset is that most of the data on GEO have been normalized. It is not surprising that ERgene had achieved this effect for a dataset that was not so well normalized.

### Further confirmed the screening results by literature

In the study of Horrison, the main purpose was to investigate the related internal reference genes for aortic lesions associated with bicuspid valve^8^. A total of 12 reference genes ATP5B, ACTB, B2M, CYC1, EIF4A2, GAPDH, SDHA, RPL13A, TOP1, UBC, YWHAZ, and 18S were detected. In his report, geNorm was used to test these 12 genes to determine the most stable single internal reference gene. We used ERgene to analyze the author’s raw data. Twenty possible reference genes were obtained (CYC16, CYC11, TOP16, CYC4, TOP11, CYC2, CYC10, CYC7, CYC12, CYC1, CYC9, EIF4A17, CYC8, TOP4, TOP10, TOP2, CYC5, TOP9, CYC3 and TOP7). All the genes that were found can divided into three families: CYC, TOP, and EIF4A. Only one member in each family should be used for normalization to not bias the results because family members may be coregulated. By comparing these three families with the 12 reference genes obtained in the study, we can see that CYC1 and TOP1 coincide, consistent with the report.

McLoughlin et al. selected a Real-Time PCR Housekeeping Gene Panel in Human Endothelial Colony Forming Cells^9^. A total of 28 candidate internal reference genes were screened out by geNorm (RPL37, RPS29, RPL9, VIM, NDUFB3, ATP51, RPL31, RPS27, CTGF, NDUPB4, ATP5J, RPS6, ACTB, ATP5FI, RPL27A, PGAM4, RPSIO, RPL30, HSPA8, RPL13, RPLI9, NDUFB8, ATP5L, UBC, VWHAC, PRDX1, GAPDH and B2M). And six stable reference genes (RPL13, RPL31, RPL37, RPL30, RPS6, and RPL19) were verified by experiments. We used ERgene to analyze the authors’ original dataset GSE125792. When the depth was set to 2, we obtained twenty candidate reference genes (RPL19, RPS4X, RPL13, TUBA1A, SF3B5, RPS3a, RPL9, RPL39L, LDHA, RPS8, RPL31, FTL, RPS3, RPL22, PINLYP, CAPG, UQCRH, RPS5, RPSAP58, and RPL36). When the depth was set to 3, seven candidate reference genes (RPL13, FTL, RPS3a, SF3B5, RPS4X, TUBA1A, and RPL19) were obtained. RPL13, the most stable reference gene determined by experiments, ranks high in our algorithm results.

### The overlap rate of genes in sample pairs

**Figure 3:**
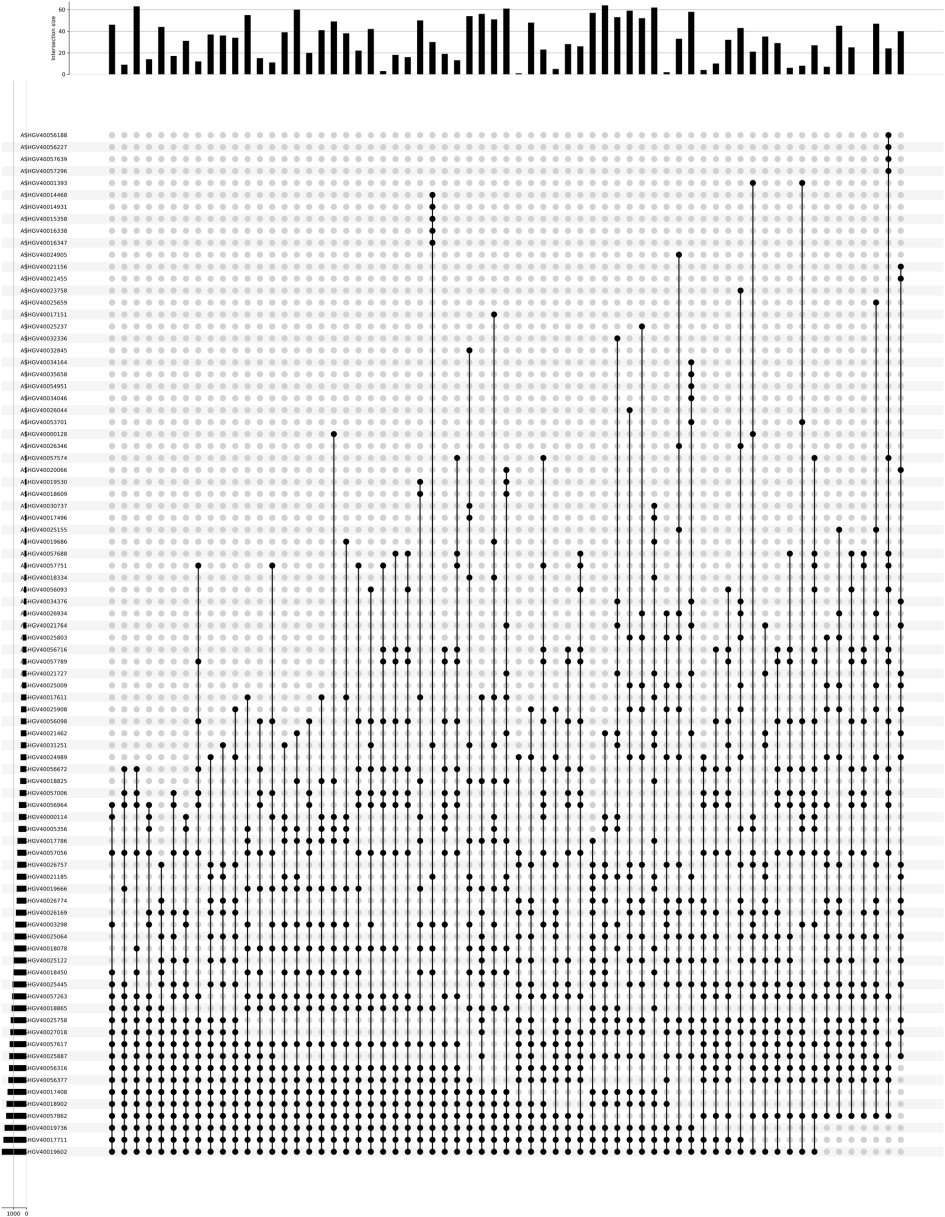
The upsetplot of 66 sample pairs overlap (GSE125792, 12 samples). The abscissa represents the sample pair, and the ordinate represents the appearance of the candidate internal reference genes. The height of the column in the upper bar chart represents the number of the sample pairs. In the upper bar chart, the height of the column represents the ordinal number of the sample pair. The higher the ordinal number, the higher the column.

ERgene screened 20 candidate internal reference genes from the two groups of samples in the dataset, and the computational depth represented the number of samples selected. When the computational depth reached 3, the 20 candidates were selected from the three pairs of samples (1, 2) (1, 3) and (2, 3), and then take the intersection. Here, we selected 12 samples of the dataset GSE125792 used by McLoughlin et al.^9^, calculated the 20 internal reference genes screened by 66 pairs of samples, and then showed the overlap of samples through the form of Upsetplot (Fig. 3). There are 10 genes in Fig. 3 with sample coverage of more than 80%. The result means that when we increase the computational depth, the duplication rate of the selected candidate internal reference gene is still more than 50%. Ten genes are included in RPL13 (probe ID: ASHGV40056316; platform: GPL21827). RPL13 was experimentally confirmed as a stable candidate internal reference gene^9^.

## Discussion

ERgene makes up for the fact that python library do not have a right method for screening reference genes, and geNorm is embedded in qbase + or Excel 2003. Normfinder is a source for the R language or Excel 2003. The application of these methods is troublesome and not particularly friendly to Python users. On the Python platform, the user only needs to enter three sentences to start filtering the internal reference gene. It seems extremely simple and friendly. The computational efficiency of ERgene is increased by nearly 90% higher than that of Normfinder, which also uses all genes for reference genes (Table 1). Besides, the internal reference genes found by ERgene, NormFinder and geNorm were similar (Table 2).

**Table 1:** Normfinder versus ERgene in computational time.

The Number of genes	2 samples		3 samples		4 samples	
	Normfinder	ERgene	Normfinder	ERgene	Normfinder	ERgene
100	0.1 s	0.1 s	0.5 s	0.66 s	1 s	1.37 s
500	35 s	0.48 s	42 s	1.35 s	44 s	2.67 s
1000	6 min	1.05 s	6 min 11 s	3.20 s	6 min 40 s	10.89 s
2000	55 min	4.58 s	55 min	10.95 s	56 min	28.47 s

**Table 2:** Comparison of internal reference genes found (the genes in bold are identical; the genes in italics are in the same family) (The test data are not converted by a probe).

	Normfinder & geNorm	ERgene
Test dataset (**No probe conversion was performed**)	P47754, **Q68FG2,** A0A0G2JDX4, Q9WVA2, P26443, Q8BU30, Q8R1Q8, Q8CBG6, Q80UW2, A0A494BAX5, **Q6PIC6**	**Q6PIC6**, A3KGU7, Q03265, P63260, O08553, Q62261, P63101, **Q68FG2**, P05064, P17182
Horrison dataset	ATP5B, ACTB, B2M, ***CYC1***, *EIF4A2*, GAPDH, SDHA, RPL13A, *TOP1*, UBC, YWHAZ	*CYC16, CYC11, TOP16, CYC4, TOP11, CYC2, CYC10, CYC7, CYC12, ****CYC1****, CYC9, EIF4A17, CYC8, TOP4, TOP10, TOP2, CYC5, TOP9, CYC3, TOP7*
McLoughlin dataset	*RPL37, RPS29, ****RPL9,*** VIM, NDUFB3, ATP51, ***RPL31****, RPS27,* CTGF, NDUPB4, ATP5J, *RPS6,* ACTB, ATP5FI, *RPL27A,* PGAM4, RPSIO, *RPL30,* HSPA8, ***RPL13****, ****RPL19***, NDUFB8, ATP5L, UBC, VWHAC, PRDX1, GAPDH, B2M	***RPL19***, *RPS4X, ****RPL13,*** TUBA1A, SF3B5, *RPS3a, ****RPL9****, RPL39L,* LDHA, *RPS8, ****RPL31,*** FTL, *RPS3, RPL22,* PINLYP, CAPG, UQCRH, *RPS5, RPSAP58, RPL36*

Although ERgene may not be new in principle, the calculation uses a new formula, which leads to a significant improvement in computing time over that of the complex matrix operations of geNorm and NormFinder. ERgene using each gene as a normalizer, calculates the ratio of each gene pair as done in the geNorm method (formula Eq. (2)). Also, the sigma squared value is equivalent to that of geNorm (formula Eq. (3)). NormFinder does not use candidate reference genes, but uses all genes to search for internal reference genes, thus, candidate instability can be avoided to a certain extent. The total number of genes tested was 1968. When the computational depth was set to 2 (screening internal reference genes with two samples) it only took the 4.58 s to obtain the possible internal reference genes because there is no complicated exponentiation. When the computational depth is set to 3 or larger, the efficiency begins to decline. When the computational depth set too large, there may be no result. Because when the computational depth is 3 or larger, the screening will select the internal reference genes from two different sample combinations, and then take the intersection from the screening results for all combinations.

The geNorm algorithm has the unique advantage of identifying the most stable reference gene from a tested set of candidate reference genes in each sample. Bestkeeper calculates all kinds of unique Bestkeeper indexes bases on the genes of the housekeeper. The amount of calculation is larger than that of geNorm, but the accuracy is improved. Both algorithms require researchers to provide genes in advance, and their applications are limited. However, ERgene directly searches and analyzes all genes according to existing samples, without the need for candidate reference genes; thus its application scope is greatly improved.

Normfinder constructed a mathematical model. It first synthesized a stable value for screening based on the intra-group variation and inter-group variation of all genes, which were improved over those of geNorm and Bestkeeper. This algorithm was excellent. ERgene used the expression multiple during internal standard normalization in the calculation of intra-group variation. And the expression multiple refered to the expression multiple of a gene relative to the internal reference gene. For example, in sample 1, if gene1 was the internal reference, gene2 should be about three times as much as gene1 in sample 2. Then the internal reference gene was screened out by the magnitude of expression multiple changes between different groups. The principle of internal reference was more consistent in ERgene than in Normfinder.

ERgene also provides a processing method for internal reference data, which is not the optimal internal reference processing method but only uses a single gene provided by ERgene. FindERG calculate the normalization factor, and the verification effect is better than other exist methods for the same experimental group. According to the MIQE guidelines^10^, it is not acceptable to normalize a single internal reference gene unless the investigator provides clear evidence to the reviewer to confirm its invariable expression under the above experimental conditions. Several studies^4^ have demonstrated the problem of using a single reference gene and recommend using at least two stably expressed reference genes. And the clustering algorithm for normalizing computing factors is most incisive in geNorm, so it is a good choice to use ERgene to screen out internal reference genes and then use geNorm or Normfinder to calculate the normalized factors for normalization processing.

## Features and methods

### Algorithms and mathematical descriptions

#### Principle

Based on internal standard method, the ratio of each gene expression quantity to the other gene expression quantity was calculated as a relative correction factor, and the calculated results were presented in the form of matrix. The ERgene algorithm inverts this process. Sample 1 calculates the relative correction factor $F$ of each gene, and sample 2 repeats the process. The differences between the results of the two samples were compared to obtain the range $\Delta F$ of the relative correction factor between each gene. ${\sigma }^{2}$ was calculated for the range of variation of each gene, then sort the results of variation from smallest to largest. The program will return the top 20 genes as a result.

When the depth is greater than two samples, for example, a depth of three samples will be selected according to the combined counting method. Three samples (1, 2), (1, 3) and (2, 3) will be selected to obtain the internal reference genes, and then the intersection will be obtained.

#### Optimization

When the gene dataset is too large, block calculation is adopted. Every 1000 genes are taken as a block. When all the blocks have been computed, the results are combined and sorted by sorting them from smallest to largest. The program will return the top 20 genes as a result.

#### Mathematical description

Let sample 1 be ${x}_{1}={\left[{A}_{1},{A}_{2},{A}_{3},\dots ,{A}_{n}\right]}^{T}$, where ${A}_{i}$ is the expression of the i-th gene in sample 1. The relative correction factor matrix ${F}_{1}$ is

1 $$\begin{aligned} F_{1} & = \left[ {x_{1} \div A_{1} ,x_{1} \div A_{2} ,x_{1} \div A_{3} , \ldots ,x_{1} \div A_{n} } \right] \\ & = \left[ {\begin{array}{*{20}c} {\frac{{A_{1} }}{{A_{1} }}} & \cdots & {\frac{{A_{n} }}{{A_{1} }}} \\ \vdots & \ddots & \vdots \\ {\frac{{A_{n} }}{{A_{1} }}} & \cdots & {\frac{{A_{n} }}{{A_{n} }}} \\ \end{array} } \right] \\ \end{aligned}$$

Similarly, let sample 2 be ${x}_{2}={\left[{B}_{1},{B}_{2},{B}_{3},\dots ,{B}_{n}\right]}^{T}$, where ${B}_{i}$ is the expression of the i-th gene in sample 2. The relative correction factor matrix ${F}_{2}$ is

2 $${F}_{2}=\left[\begin{array}{ccc}\frac{{B}_{1}}{{B}_{1}}& \cdots & \frac{{B}_{n}}{{B}_{1}}\\ \vdots & \ddots & \vdots \\ \frac{{B}_{n}}{{B}_{1}}& \cdots & \frac{{B}_{n}}{{B}_{n}}\end{array}\right]$$

The relative factor change amplitude matrix $\Delta F$ is

3 $$\Delta F={F}_{1}-{F}_{2}=\left[\begin{array}{ccc}\frac{{A}_{1}}{{A}_{1}}-\frac{{B}_{1}}{{B}_{1}}& \cdots & \frac{{A}_{n}}{{A}_{1}}-\frac{{B}_{n}}{{B}_{1}}\\ \vdots & \ddots & \vdots \\ \frac{{A}_{n}}{{A}_{1}}-\frac{{B}_{n}}{{B}_{1}}& \cdots & \frac{{A}_{n}}{{A}_{n}}-\frac{{B}_{n}}{{B}_{n}}\end{array}\right]$$

The variance vector ${\sigma }^{2}$ of the magnitude of change in relative factors for each gene is

4 $${\sigma }^{2}={\left[\begin{array}{ccc}\frac{\sum {(\Delta {F}_{1i}-\frac{\sum ({\Delta F}_{1i})}{n})}^{2}}{n}& \dots & \frac{\sum {(\Delta {F}_{ni}-\frac{\sum ({\Delta F}_{ni})}{n})}^{2}}{n}\end{array}\right]}^{T}$$

### Function description

#### ERgene.FindERG(data, depth)

This function is used to screen internal reference genes. The parameter data are in DataFrame format, where the first column is the gene ID, and the other column is the expression level of each gene in the sample. The depth of the parameter refers to the number of samples to be selected for internal reference genes. For example, a depth of 2 is used for screening samples 1 and 2. A depth of 3 means sample 1 and sample 2, sample 1 and sample 3, and sample 2 and sample 3 are screened separately, and then the intersection is removed. The greater the depth, the fewer the number of internal reference genes screened, which cannot be fewer than 2 or more than the number of samples. And users can compare the results at different depths. The speed of calculation depends on the depth of calculation. Genes in calculation results may come from the same family, and only one family member should be used in normalization. When the calculation depth is larger than 2, an Upsetplot will be generated to show the overlap rate of the candidate internal reference genes generated by each pair of samples (Fig. 3).

#### ERgene.normalizationdata(data, ERGname)

This function is used for the standardization of a single internal reference gene. The parameter data are in DataFrame format, where the first column is the gene ID, and the second column is the expression level of each gene in the sample. The parameter ERGname is the name of the internal reference gene to be processed. The computation speed is accelerated by using the multi-threaded matrix operation of Pandas, making the computation speed faster.

## Data Availability

Raw test data are available at https://github.com/Starlitnightly/ERgene/tree/master/example. The GEO datasets analyzed during the current study are available in the Gene Expression Omnibus repository, https://www.ncbi.nlm.nih.gov/geo/query/acc.cgi?acc=GSE4786, https://www.ncbi.nlm.nih.gov/geo/query/acc.cgi?acc=GSE125792.

## Code availability

Source code is available for academic non-commercial research purposes. Links to code and documentation are provided at https://github.com/Starlitnightly/ERgene.
